# Variable effects of non-falciparum species infections on malaria disease severity in high transmission regions in Senegal

**DOI:** 10.1186/s41182-024-00655-8

**Published:** 2024-12-04

**Authors:** Aissatou Diagne, Babacar Souleymane Sambe, Folly Mawulolo Gaba, Ibrahima Sarr, Arona Sabène Diatta, Ousmane Sadio, Serigne Ousmane Mbacké Diaw, Hélène Ataume Mawounge Diatta, Babacar Diouf, Inès Vigan-Womas, Babacar Mbengue, Makhtar Niang

**Affiliations:** 1 Institut Pasteur de Dakar, Pôle Immunophysiopathologie et Maladies Infectieuses, 220 Dakar, Senegal; 2 Université Cheikh Anta Diop de Dakar, Service d’Immunologie FMPO, Dakar, Senegal

**Keywords:** Malaria, *Plasmodium* species, Diagnostic, Disease severity

## Abstract

In malaria endemic countries, non-falciparum species are often mixed with *Plasmodium falciparum* in patients with uncomplicated malaria, and their contribution to malaria severity and death is poorly studied. This study assesses the contribution of non-falciparum species to malaria severity in three regions of Senegal with the highest malaria incidence.

We analysed 617 blood samples obtained between 2015 and 2021 from confirmed malaria patients at health facilities in Kedougou, Kolda and Tambacounda in Senegal. *Plasmodium* species composition was determined by PCR and their distribution were analysed according to age and disease severity, and the relative risk of developing severe malaria.

Overall, 94.8% of samples contained *P. falciparum* either as single or mixed with other species. Non-falciparum *P. ovale, P. vivax* and *P. malariae* species were detected in 60.12, 13.61 and 1.62% of samples, respectively. Severe malaria was primarily due to *P. falciparum*, but co-infection with *P. vivax* led to a 1.63-fold significant (*p* = 0.05) increased risk of developing severe malaria, contrasting with the non-significant reduced risk (OR = 0.78; CI 95: 0.55–1.11; *p* = 0.16) associated with *P. ovale* infections. Children aged < 15 years old significantly suffered of SM than adults patients, whereas no significant association was found in relation to patient’ sex.

This study reports the first association of non-falciparum species infections with clinical malaria phenotypes in patients from the three most malaria-affected regions in Senegal. Non-falciparum *P. ovale* and *P. vivax* species in combination with *P. falciparum* had a protective and worsening effect, respectively. The findings suggest that interventions targeting only *P. falciparum* might not be sufficient to eliminate the overall malaria burden, and should take into account the neglected non-falciparum species.

## Introduction

Malaria remains an important parasitic disease with more than half of the world's population exposed to the disease. Since early 2000s, excluding the COVID-19 pandemic years, a steady decline of the number of cases and deaths attributed to malaria has been reported in many places. The World Health Organization (WHO) reported a rapid decline of malaria incidence by 27% between 2015 and 2000, which was slower (less than 2%) during the 2015–2019 period [1].

To date, the infecting *Plasmodium* parasite species, the levels of innate and acquired immunity of the human-infected host, and the timing and efficacy of treatment are all known factors that influence the clinical manifestations of the infection and the likelihood of its progression from an asymptomatic state to uncomplicated (UM) or severe malaria (SM), and ultimately to death [2]. Research on malaria severity is largely centred on *P. falciparum* because of its evident role in the burden of the disease [3], contrasting with the lesser research on non-falciparum species despite their evident co-existence at variable levels with *P. falciparum* in areas where these species were previously thought to be absent.

In Senegal, studies have revealed the presence of four human infecting *Plasmodium* species (i.e., *P. falciparum*, *P. ovale*, *P. malaria*e and *P. vivax*) in samples from febrile patients and/or asymptomatic individuals [4–8], though to date, only *P. falciparum* has been formally associated with the severity of the disease. In the vast majority of cases, non-falciparum species are documented in mixed infections with the dominant *P. falciparum* species at various proportions in given areas [4, 6, 9, 10] but no real prevalence data is available nationwide. Due to their low prevalence and the low risk to cause severe malaria, non-falciparum species are neglected in research and malaria control strategies in Senegal and their potential contribution to severe malaria has not been assessed. However, studies have shown that even at low parasitaemia, infections with non-falciparum species can lead to severe forms of malaria and even death [11–14].

This study investigates the potential contribution of non-falciparum species on the severity of malaria in patients from the three regions of Senegal with the highest incidence of malaria [15].

## Materials and methods

### Study sites

**Fig. 1:**
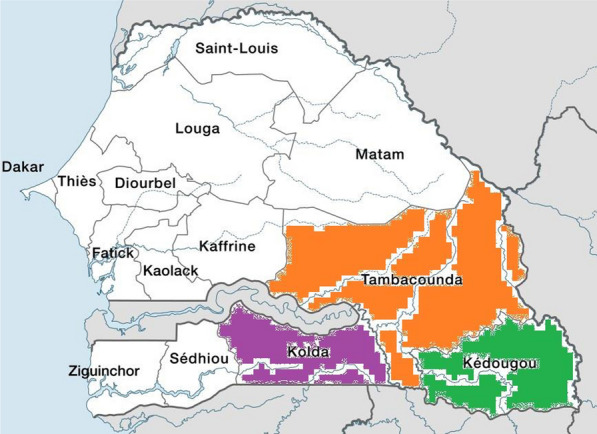
Map of Senegal showing the three study regions in the South and southeast Senegal

Patient’s samples used in this study were obtained in 2015, 2017, 2020 and 2021 from the regional hospitals of Kolda, Tambacounda, and Kedougou, respectively situated in the south and south-east of Senegal (Fig. 1). The three regions cover 37% of the national territory with a demographic weight of 11.3% [16]. They are of epidemiological importance due to the very high incidence of malaria since the three regions account together for 78.5% of the confirmed clinical malaria cases in Senegal in 2021 [15]. Their geographical location on the borders with Mali, Gambia and Guinea made them subjected to a strong flow of people that could contribute to the importation of *Plasmodium* parasites and the maintenance of transmission.

### Study population and sampling

The study included febrile patients who consulted in the regional hospitals of Kolda in 2015, Tambacounda in 2017, 2020 and 2021, and Kedougou in 2020 and 2021. All patients aged 6 months or older and presenting with fever (axillary temperature > 37.5 °C) and one or more symptoms suggestive of malaria disease (headache, nausea, dizziness, chills, fatigue, etc.) were approached for their consent or assent and their inclusion in the study.

The minimum sample size for the study, with a 95% confidence interval and a precision of 1%, was calculated using the following formula:

$$n =\frac{1.96 \times p \times (1 -p)}{{e}^{2}}$$

*n*: sample size; *p*: prevalence of rarest *Plasmodium* species (we estimated a prevalence of 1.5% based on our surveillance work on non-falciparum species, which generally shows that the prevalence of *P. vivax* and *P. malariae* ranges between 1 and 3%); *e*: precision.

Taking together all these parameters (confidence interval of 95%, *p* = 1.5% and *e* = 1%), the minimum sample size required for this study is approximately 568 individuals.

For each enrolled patient, venous blood was collected on an EDTA tube for molecular diagnosis of *Plasmodium* infection and the characterization of *Plasmodium* species. Demographic and clinical data of patients such as age, sex and disease severity were recorded in a dedicated register.

In the absence of a pyrogenic threshold within the country, the classification of malaria into severe and uncomplicated cases was determined by clinicians as follows: (i) the uncomplicated malaria group (UM) comprized patients who tested positive for the *Plasmodium* genus and exhibit the following symptoms during the time of consultation: fever, headache, chills, vomiting, myalgia, anorexia, and sweating, in the absence of any identifiable alternative cause; (ii) the severe malaria group (SM) consisted of patients who were *Plasmodium* genus positive with symptoms of uncomplicated malaria along with at least one acute failure in the following functions: neurological, hemodynamic, respiratory, renal, hepatic, haematological, or metabolic. This classification was made in the absence of any identifiable alternative cause [1].

### Molecular detection and characterization of *Plasmodium* species

Genomic DNA (gDNA) of *Plasmodium* parasites was isolated from blood using the commercially available QIAamp DNA Blood Mini Kit (Qiagen, Hilden, Germany) according to manufacturer’s instructions with minor modifications. Two hundred microliters (200 μl) of blood pellet were used for extraction and final elution of isolated DNA was performed in 50 μl volume. DNA was stored at − 20 °C until further analysis.

The presence of *Plasmodium* parasites was first investigated by a “screening real-time quantitative PCR” (qPCR) as previously described by Niang et al. [17] with genus-specific primers targeting the *Plasmodium cytochrome b* gene. In a second assay, a species-specific nested PCR was used on qPCR positive DNA samples to differentiate the four major *Plasmodium* species i.e. *P. falciparum*, *P. vivax*, *P. malariae* and *P. ovale* according to the protocol described by Snounou et al. [18]. The gDNA from confirmed *P. falciparum*, *P. malariae*, *P. ovale* and *P. vivax* infected blood samples served for positive controls in all amplifications; sterile water and gDNA from uninfected blood samples served for negative controls to ensure lack of contamination. Nested PCR results were scored as categorical variables (presence vs absence of amplification).

### Statistical analysis

The data was analyzed using R version 4.1.017. A descriptive analysis was performed to examine the distribution of individuals based on age, sex, study site, study period, and clinical phenotype (UM and SM). For some analyses, death cases were considered as a specific group. The association of non-falciparum species with disease severity was assessed using binomial logistic multi-variate regression, with age, sex, and the presence or absence of non-falciparum species as explanatory co-factors. Due to the low prevalence of unique non-falciparum infections, the logistic regression only included *P. falciparum*-positive individuals (in single or multiple infections). The level of statistical significance was set at 5% (*p*-value is significant if it is less than or equal to 0.05). The p-value of the logistic regression model was calculated without and with adjustment using the Benjamini & Hochberg (B&H) method.

## Results

### Baseline characteristics of the study population

A total of 617 samples from *Plasmodium*-infected patients confirmed by PfRDT and/or microscopy, and qPCR were included in this study (Table 1). Among the 617 patients, 77 were recruited from Kolda in 2015, 306 from Tambacounda (90 in 2017, 83 in 2020, and 133 in 2021) and 234 from Kedougou (23 in 2020 and 211 in 2021). Except in Tambacounda in 2020, there were as many male as female patients (Table 1). Age of patients ranged from 0.5 to 81 years old (Table 1), but the mean age varied between regions and sampling period, and was highest and lowest in Kolda’s patients (24.79 ± 2.52) and Tambacounda 2017 (10.43 ± 1.37), respectively (Table 1). The majority of patients recruited from Kolda and Kedougou were over 15 years of age (Table 1).

**Table 1:** Baseline characteristic of the study population

Sites	Kolda	Tambacounda			Kedougou	
Years	2015	2017	2020	2021	2020	2021
Effective	77	90	83	133	23	211
Age (years)						
NA’s	0	0	0	1	0	0
Mean	24.79	10.43	11.07	11.88	20.87	20.82
SE	2.52	1.37	1.16	1.13	3.34	1.04
Median	18	6	8	7	16	18
Range	0.91–77	0.5–65	1.17–55	0.75–81	2.08–70	0.67–72
Age groups (%)						
NA’s	0	0	0	1	0	0
0–5 years	29.87	42.22	36.14	37.88	4.35	15.64
5–15 years	10.39	43.33	45.78	35.61	39.13	22.75
+ 15 years	59.74	14.44	18.07	26.52	56.52	61.61
Sex (%)						
NA’s	0	0	0	0	0	0
Female	49.35	48.89	30.12	51.13	43.48	49.29
Male	50.65	51.11	69.88	48.87	56.52	50.71
Ratio M/F	1.03	1.05	2.32	0.96	1.3	1.03

*NA’s* Number of missing data, *M* Male, *F* Female, *SE* Standard Error

### Distribution of clinical malaria according to sex and age of patients

The proportions of patients presenting with UM and SM phenotypes were analysed in relation to patients’ origin, gender, and age (Table 2). Both UM and SM patients originated in majority from Tambacounda and represented 78.8 and 67.68%, respectively (Table 2). Similarly, 93.3% (14/15) of deaths were recorded in Tambacounda (Table 2). In each clinical phenotype, the proportions of male and female were similar, while recorded death was 2.5 higher in male than female (Table 2). The mean age of UM patients was higher than that of SM patients (18.36 vs 13.82), but the age range were comparable (Table 2). While children and older individuals were evenly represented in UM patients, young children and adolescents aged 0–15 years old were the most affected by SM infection as they represented together 70.78% of the SM patients (Table 2). The separate analysis of the death group indicated that 93.34% of deaths occurred in younger children and adolescents (Table 2).

**Table 2:** Clinical phenotype distribution according to age, sex and site

	UM (*n* = 373)	SM (*n* = 244)	Death (*n* = 15)
Site (year)			
NA’s	0	0	0
Kolda (2015)	19	57	1
Tambacounda (2017)	66	20	4
Tambacounda (2020)	42	41	0
Tambacounda (2021)	29	94	10
Kedougou (2020)	12	11	0
Kedougou (2021)	205	6	0
Sex (%)			
NA’s	0	0	0
Female	46.52	47.33	26.67
Male	53.48	52.67	73.33
Ratio M/F	1.16	1.11	2.5
Age (years)			
NA’s	1	0	0
Mean (SE)	18.36	13.82	6.33
SE	0.82	0.99	1.52
Median	15	8	6
Range	0.5–77	0.75–81	1.08–23
Age group (%)			
NA’s	1	0	0
0–5 years	23.59	35.80	46.67
5–15 years	27.88	34.98	46.67
+ 15 years	48.53	29.22	6.66

*UM* Uncomplicated malaria, *SM* Severe malaria (including death), *NA’s* Number of missing data, *M* Male, *F* Female, *SE* Standard Error

### Frequencies of clinical malaria phenotype according to *Plasmodium species* infections

Regardless of the type of infection (single or mixed) or the clinical phenotype (UM or SM), *P. falciparum* was detected in 94.8% (585/617) of overall infections (Table 3). Non-falciparum species either as single or mixed were detected in both clinical groups. In non-falciparum infections *P. ovale* was the dominant species, present in 84.66 and 89.58% of SM and UM patients (Table 3). *Plasmodium vivax* were present in 26.99 and 15.44% of patients with SM and UM, respectively (Table 3). Although present in both UM and SM samples, *P. malariae* was exclusively seen in co-infections with other species, and was the least represented among the non-falciparum species at respectively 3.68 and 1.54% in patients with SM and UM (Table 3).

**Table 3:** Clinical phenotype according to the type of infections (falciparum or non-falciparum infections)

	Clinical phenotype	
	SM	UM
	*n* (%)	*n* (%)
Falciparum infections (*n* = 585)	232	353
Single	80 (34.48)	115 (32.58)
Mixed	152 (65.52)	238 (67.42)
Non-falciparum infections* (*n* = 422)	163	259
Po	138 (84.66)	232 (89.58)
Pv	44 (26.99)	40 (15.44)
Pm	6 (3.68)	4 (1.54)

*UM* Uncomplicated malaria; *SM* Severe malaria (including death)

^*^Numbers include co-infections between species; Po: *P. ovale*; Pv: *P. vivax*; Pm: *P. malariae*

**Fig. 2:**
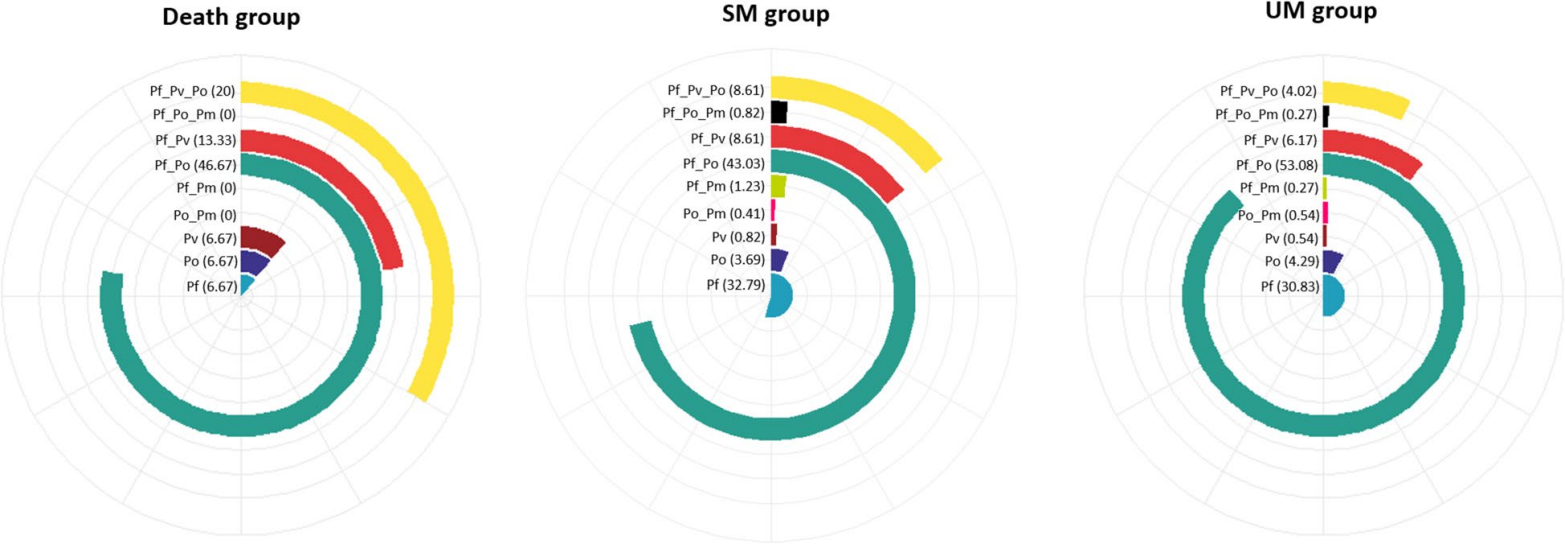
*Plasmodium* species distribution. *death* death group, *SM* Severe malaria group (excluding death) , *UM* Uncomplicated malaria group, *Pf* *P. falciparum;* *Po P. ovale*, *Pv P. vivax*, *Pm P. malariae*

Detailed analysis of *Plasmodium* species composition in the study groups revealed the presence of unique non-falciparum infections in all clinical groups at various proportions as well as high proportions of mixed *P. falciparum/P. ovale* and *P. falciparum/vivax* in UM (53.08 and 6.17%) and SM (43.03 and 8.61%) (Fig. 2). These *P. falciparum/P. ovale* and *P. falciparum/vivax* infections were even found in death cases with proportions of 46.67 and 13.33% respectively (Fig. 2). Though all four *Plasmodium* species are known associated with UM, the study found single non-falciparum species, notably *P. vivax* and *P. ovale* in SM and deaths patients groups (Fig. 2).

### Relative risk of developing severe malaria according to the type of infection

**Fig. 3:**
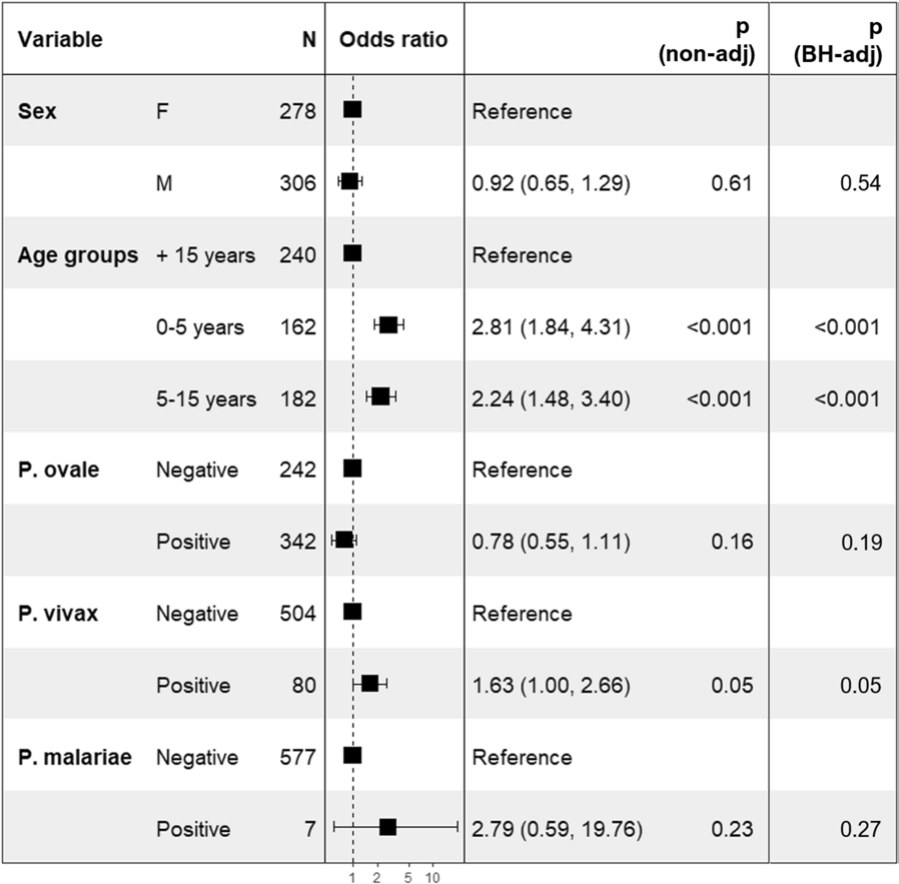
Severe malaria relative risk assessment in the presence of non-falciparum species

The individual datasets from single and mixed *Plasmodium* species identified in SM patient’s samples (including those of deceased patients) as well as patient’s age and sex were used to assess the relative risk of malaria severity. No significant association was found between patient’ sex and the severity of the disease, whereas younger (0–5 years) and older children (5–15 years) significantly suffered from SM than adult’s patients (Fig. 3). In the presence of *P. falciparum*, additional infection with *P. ovale* reduced though non-significant, the relative risk of SM (OR = 0.78; CI 95 = 0.55–1.11; *p* = 0.16), but co-infections with *P. vivax* led to 1.63 significant fold increase of the relative risk of SM (CI 1–2.26; *p* = 0.05).

## Discussion

The COVID-19 pandemic has had a considerable impact on strategies to control infectious diseases, particularly malaria. As a result, a slight resurgence of malaria cases was observed worldwide [19]. Indeed, in health facilities in Senegal and elsewhere in the world, not only COVID-19 patients were prioritized for care, but also febrile individuals feared to report to health facilities. This could explain the increase in the proportions of SM cases observed among children (0–15 years) in 2020 and 2021 in Tambacounda and Kedougou regions. Indeed, compared to 2017 during which there were thrice more UM cases than SM cases, 0–15 years old patients presenting with SM were highest in 2020. The high proportion of SM patients obtained in Kolda in 2015 was due to the fact that the sampling targeted primarily SM cases from hospitalized patients.

The high burden of *P. falciparum* malaria in many African countries reasonably justifies the focus of malaria research and interventions on *P. falciparum*, and the little effort on non-falciparum species [19]. However, the increased prevalence of non-falciparum malaria, notably *P. vivax* in many endemic areas, including in West Africa call upon a reconsideration of non-falciparum malaria as an emerging problem in Africa. In Senegal, the recent reports of *P. vivax* infections in asymptomatic and febrile patients in the Kedougou region [7, 8], and the established transmission of *P. ovale* and *P. malariae* across the country [6, 7, 20, 21] warrant more attention before the country could fully commit to malaria elimination.

The present study provides additional information on single and mixed *Plasmodium* species infections among febrile patients living in the three most malaria-affected Senegalese regions, and the potential contribution of non-falciparum species in the outcome of the disease. Non-falciparum species are recurrently reported in Senegal by various research groups and at different periods [6–8, 20–22]. Due to their biological particularities, most of the current control strategies directed towards *P. falciparum* are not suitable against non-falciparum species [23–25], thus favouring their endemicity. Moreover, in many settings where *P. falciparum* and other *Plasmodium* spp. are co-endemic, a relative increase of non-falciparum species infections is often revealed when the interventions against *P. falciparum* have become successful [26].

To date, the role of *P. falciparum* in malaria disease severity and malaria-related deaths is no longer a debate, and the high frequencies of single *P. falciparum* species in all the three clinical phenotypes investigated in this study sufficiently confirm *P. falciparum* as the primary cause of SM and malaria deaths. However, the presence of single *P. vivax* and *P. ovale* infections in both UM and SM cases strongly suggest a role in malaria morbidity. In fact, in addition to their relatively high frequency in UM patients, the frequencies of single *P. vivax* and *P. ovale* infections in patients with SM argue for an implication of the two neglected *Plasmodium* species in the severity of the disease. To the best of our knowledge, this study reports the first unique presence of non-falciparum species in SM patients in high transmission regions in Senegal. Elsewhere, neglected non-falciparum species have been implicated in severe malaria cases or death [12–14, 27].

The current study revealed that *P. vivax* has a significant aggravating contribution to the occurrence of SM, a finding that is in line with the review by Naing et al. [28] which reported that the incidence of severe malaria in patients infected by *P. vivax* was considerable following a review of 26 studies that analysed the involvement of *P. vivax* and *P. falciparum* in the severity of malaria [28].

In a study that examined peripheral blood parasitemia of vivax-infected patients (53 UM and 9 SM), along with falciparum-infected patients (109 UM and 22 SM), Barber et al. [29] and Baird [30] concluded that peripheral parasitemia underestimated pathogenic biomass, especially in severe disease [29]. Indeed, *P. vivax* has a tropism for extravascular tissues in the bone marrow and spleen, which explains why low levels or undetectable parasitemia in peripheral blood can result in both acute and chronic disease [30]. In addition, Silva-Filho et al. [31] reported a six-fold higher peripheral parasitemia in severe than non-severe *P. vivax*-infected patients [31]. Our recent molecular detection and quantification of *P. vivax* in patient’s samples has revealed the low-level of peripheral *P. vivax* parasitemia [32], thus confirming the challenges to detect *P. vivax* by RDT and reference microscopy methods. Accordingly, hidden *P. vivax* parasitemia could induce anemia which has been identified by Douglas et al. as the most common cause of death attributable to infection by *P. vivax* at a hospital in eastern Indonesia [33]. Moreover, in patients, especially young children co-infected by *P. falciparum* and *P. vivax*, an exacerbation of anemia has been pointed out [34].

By contrast, *P. ovale* appears to have a protective effect against severe malaria in our study. The finding is in line with the data reported by Kotepui et al. in a meta-analysis of eight studies that include a total of 1,365 cases of malaria with *P. ovale* [35]. Similarly, the implication of *P. ovale* in fever episodes has been clearly established in Senegal both in children and adults, but the incidence of the disease was much lower than for *P. falciparum* [20, 36].

Concerning *P. malariae*, its contribution to SM could not be clearly elucidated in the current study because of its absence as single infection in SM patients and its very low prevalence in mixed infections with *P. falciparum* and *P. ovale*; though other studies have implicated *P. malariae* to cases of severe malaria [27]. In the global literature, there is little evidence that *P. malariae* may be responsible for fever episodes in children or adults living in high malaria endemic areas, but the occurrence of fever episodes associated to *P. malariae* parasitemia was reported in Nigeria [37]. The 2.79-fold increase of SM associated with *P. falciparum*/*P. malariae* infections found in our study was not significant and could be associated with the very low number of *P. malariae* cases.

Two of Senegal's neighbouring countries, Mali and Mauritania, are respectively homes of moderate and high incidences of *P. vivax* malaria in parts of their countries [38, 39]. To progress towards the pre-elimination phase, Senegal must address the worrying emergence of *P. vivax* malaria in the Kedougou and neighbouring regions of Kolda and Tambacounda and the parasite’s presence in UM and SM patients, along with the established transmission of *P. ovale* and *P. malariae* throughout the country.

## Conclusion

The study presents unique non-falciparum species infections in patients with severe malaria with a variable implication of *P. vivax* and *P. ovale* in the severity of the disease relative to *P. falciparum*. The increasing report of single non-falciparum infections in UM and SM patients from the three regions of Senegal with the highest incidence of malaria suggest that non-falciparum malaria should no longer be neglected and must be targeted with evidence-based and concerted interventions if the ambiguous goal of elimination is to be achieved.

## Data Availability

All relevant data are within the paper. Specific datasets are available from the corresponding author on reasonable request.
